# Leukemias in the Context of Rheumatoid Arthritis: Shared Pathways and Clinical Perspectives

**DOI:** 10.7759/cureus.93082

**Published:** 2025-09-24

**Authors:** Alaa Osman, Nipun Addla, Ashesh Das, Badriya Ali Alansari, Divya Iyer, Maria Jose Acosta, Khaled Aldhuaina, Aakriti Datta, Anandhu Anil Nair, Manju Rai

**Affiliations:** 1 Medicine, Lebanese University, Beirut, LBN; 2 Internal Medicine, Kamineni Academy of Medical Sciences and Research Centre, Hyderabad, IND; 3 Internal Medicine, Kali Pradip Chaudhuri (KPC) Medical College and Hospital, Kolkata, IND; 4 Medicine, Gulf Medical University, Ajman, ARE; 5 Internal Medicine, University College Dublin (UCD) School of Medicine, Dublin, IRL; 6 Internal Medicine, Universidad del Rosario, Bogota, COL; 7 Internal Medicine, Faculty of Medicine, Kuwait University, Kuwait City, KWT; 8 Internal Medicine, Kasturba Medical College, Manipal, IND; 9 Internal Medicine, Amrita Institute of Medical Sciences, Kochi, IND; 10 Biotechnology, Shri Venkateshwara University, Gajraula, IND

**Keywords:** chronic inflammation, cytokines, genetic predisposition, hematological malignancies, il-6, immune dysregulation, leukemia, rheumatoid arthritis, risk stratification, tnf-alpha

## Abstract

Rheumatoid arthritis (RA) is a chronic autoimmune disorder characterized by systemic inflammation, progressive joint destruction, and increased risk of malignancies, particularly hematological cancers such as leukemias. RA patients appear to have a higher incidence of leukemias, suggesting a possible association between the two conditions. This association is driven by shared pathogenic mechanisms, including chronic inflammation, immune dysregulation, and genetic predispositions. Pro-inflammatory cytokines, notably tumor necrosis factor-alpha (TNF-α) and interleukin 6 (IL-6), play a crucial role in sustaining an inflammatory microenvironment that promotes leukemic transformation. Genetic alterations, including mutations in STAT3, TET2, and DNMT3A, further highlight the overlap between RA pathophysiology and hematologic malignancies. Moreover, RA treatments such as methotrexate (MTX), Janus kinase (JAK) inhibitors, and anti-TNF therapies have complex implications, with some studies suggesting potential contributions to leukemia risk through immune suppression and hematopoietic alterations. Clinical implications of this association emphasize the necessity of early detection strategies, biomarker-based risk stratification, and close hematologic monitoring in RA patients. Interdisciplinary collaboration between rheumatologists and hematologists is essential for optimizing treatment approaches while minimizing oncogenic risks. Future research should focus on identifying predictive biomarkers, exploring targeted therapeutic interventions, and elucidating the molecular mechanisms underlying RA-associated leukemias. Advances in multiomics and artificial intelligence-driven risk modeling may facilitate personalized treatment strategies, improving both RA management and leukemia prevention. Given the rising burden of RA and its associated complications, a comprehensive understanding of its link to leukemias is critical for enhancing patient outcomes and guiding clinical decision-making.

## Introduction and background

Rheumatoid arthritis (RA) is a complex chronic inflammatory autoimmune disease characterized by symmetric polyarthritis, progressive joint destruction, and systemic manifestations affecting approximately 0.5-1% of the global adult population [1]. The disease is marked by persistent synovial inflammation, autoantibody production, and dysregulated immune responses, leading to significant morbidity and reduced quality of life [2]. Beyond joint manifestations, RA is associated with various extra-articular complications and an increased risk of malignancies, particularly hematological disorders.

Epidemiological studies have consistently demonstrated a higher incidence of hematological malignancies, especially lymphomas and leukemias, in RA patients compared to the general population [3-4]. Parikh-Patel et al. reported a standardized incidence ratio (SIR) for leukemia in RA patients ranging from 1.5 to 2.4, varying by population characteristics [5]. In the same cohort, the incidence rate (IR) of leukemia among RA patients was estimated at 0.11 per 1,000 person-years, providing a clinically meaningful measure of absolute risk. This increased risk has been attributed to multiple factors, including chronic inflammation, immune system dysregulation, and the potential effects of immunosuppressive treatments commonly used in RA management [6].

Recent advances in molecular biology and immunology have revealed intriguing parallels between the pathogenic mechanisms underlying both RA and leukemias. These diseases share several molecular pathways involving cytokine networks, cellular signaling cascades, and immune system regulation [7]. Understanding these shared pathways is crucial for both diseases' prevention, early detection, and management strategies.

This review seeks to provide an in-depth analysis of the intricate relationship between RA and leukemias, focusing on three key objectives. First, it examines the shared molecular and cellular mechanisms underlying both conditions, including inflammatory mediators, immune dysfunction, and genetic predispositions. Second, it evaluates the potential influence of RA treatments on leukemia risk and progression. Finally, it addresses the clinical implications of these findings, highlighting their relevance for patient monitoring, risk stratification, and informed therapeutic decision-making in individuals with RA.

## Review

Search methodology

A comprehensive literature search was conducted to identify studies examining the relationship between RA and leukemias. The search was performed across major electronic databases, including PubMed/MEDLINE, Scopus, Embase, and Web of Science up to July 2025, with additional references identified through manual screening of bibliographies of relevant articles. The search strategy combined medical subject headings (MeSH) and free-text terms. The primary MeSH terms included “Rheumatoid Arthritis,” “Leukemia,” “Lymphoproliferative Disorders,” “Hematologic Neoplasms,” “Cytokines,” and “Immune Dysregulation.” Free-text keywords such as “RA-associated leukemia,” “rheumatoid arthritis and hematological malignancies,” “immune dysregulation in RA and leukemia,” “methotrexate-induced leukemia,” and “JAK-STAT pathway in RA and leukemia” were also applied. Boolean operators were used to combine terms, for example, “Rheumatoid Arthritis AND Leukemia,” “RA AND hematologic malignancy,” and “RA AND genetic mutations.”

Studies were included if they were original research (observational, cohort, case-control, or clinical trials), systematic reviews, meta-analyses, or narrative reviews addressing leukemias in the context of RA. Only articles published in English were considered. Exclusion criteria included case reports or small case series with fewer than five patients, non-English publications, conference abstracts, editorials, or commentaries without substantive data, and studies focusing exclusively on malignancies other than leukemia unless they provided mechanistic or comparative insights relevant to leukemogenesis. Two reviewers independently screened titles and abstracts for relevance, followed by full-text evaluation of potentially eligible studies, with discrepancies resolved by consensus. The final selection prioritized the most relevant and high-quality studies to ensure a balanced synthesis of current evidence.

RA pathophysiology and immune dysregulation

RA pathogenesis involves complex interactions between genetic predisposition, environmental triggers, and dysregulated immune responses. The disease process is initiated when self-tolerance breaks down, leading to the activation of both innate and adaptive immune responses against joint tissues [8]. This autoimmune response is characterized by the production of autoantibodies, particularly rheumatoid factor (RF) and anti-citrullinated protein antibodies (ACPAs), which form immune complexes and perpetuate inflammation [9].

The synovial membrane in RA undergoes significant changes, with hyperplasia of synovial fibroblasts and infiltration of immune cells creating an aggressive, tumorlike tissue called pannus. T cells, particularly CD4+ helper T cells, play a central role by secreting pro-inflammatory cytokines and activating other immune cells [10]. The T helper 17 (Th17) subset has emerged as a key player, producing IL-17 and other inflammatory mediators that promote joint destruction. B cells contribute to pathogenesis through multiple mechanisms, including autoantibody production, antigen presentation, and cytokine secretion [11-13].

Genetic studies have identified over 100 risk loci associated with RA, with HLA-DRB1 shared epitope alleles conferring the strongest genetic risk [14]. These genetic factors interact with environmental triggers, including smoking, periodontitis, and certain infections, which can initiate disease in susceptible individuals [15]. Epigenetic modifications, particularly DNA methylation and histone modifications, provide an additional layer of regulation and may explain how environmental factors influence disease development [16].

Leukemias: types, risk factors, and pathophysiology 

Leukemias represent a group of hematological malignancies defined by uncontrolled white blood cell proliferation, encompassing four primary categories: acute myeloid leukemia (AML), chronic myeloid leukemia (CML), acute lymphoblastic leukemia (ALL), and chronic lymphocytic leukemia (CLL). These distinct variants demonstrate unique molecular and clinical features that guide therapeutic strategies and outcome predictions [17-18]. While acute forms (AML and ALL) demand immediate treatment due to their aggressive progression, chronic variants (CML and CLL) typically begin with observation-based management [18-19].

The pathogenesis of leukemias involves intricate interactions among immune system regulation, genetic predisposition, and environmental factors [18,20]. Immune system disruption plays a particularly significant role in CLL development, where it triggers substantial alterations in immune cell distributions [21,22]. Specifically, CLL leads to the replacement of functional CD8+ cytotoxic and CD4+ activated effector T cells with exhausted and immunosuppressive variants, thereby compromising anti-tumor immunity [18,23].

The inflammatory environment in RA contributes to leukemic transformation through sustained cytokine imbalance and T cell dysfunction [18,24-25]. Elevated levels of pro-inflammatory cytokines, particularly tumor necrosis factor-alpha (TNF-α), interleukin-6 (IL-6), and interleukin-17 (IL-17), are fundamental to both RA and leukemia progression [24]. TNF-α promotes leukemic cell survival, proliferation, and angiogenesis by activating anti-apoptotic and inflammatory cascades. IL-6, acting through the JAK-STAT3 pathway, facilitates proliferation and resistance to apoptosis, while IL-17 stimulates neutrophil recruitment, supports leukemic microenvironmental changes, and maintains a pro-inflammatory state [25-26]. IL-17A, a hallmark Th17 cytokine, has been shown to promote proliferation and chemoresistance in Philadelphia chromosome-positive B-ALL through activation of JAK-STAT3 and NF-κB pathways, while also modulating the bone marrow niche to favor leukemic persistence [27]. Other Th17-related cytokines, such as IL-21 and IL-22, further support leukemogenesis by remodeling the tumor microenvironment and activating pro-survival pathways like STAT3 and AKT [26,28]. These findings suggest a broader and more complex role for Th1/Th17 cytokines in the immunopathogenesis of RA-associated leukemias. These cytokines exert their pro-leukemic effects via activation of key intracellular signaling pathways: the Janus kinase/signal transducer and activator of transcription (JAK-STAT) and nuclear factor-kappa B (NF-κB) pathways [29]. JAK-STAT activation, especially involving STAT3, drives oncogenic transcription programs, enhances survival signals, and supports chronic inflammation [24]. Simultaneously, the NF-κB pathway modulates transcription of genes responsible for cytokine production, cell survival, immune evasion, and hematopoietic deregulation. Persistent stimulation of these pathways disrupts immune surveillance, alters hematopoietic niches, and facilitates malignant transformation.

The dysregulated cytokine milieu also creates unfavorable bone marrow conditions by triggering precursor cell death and clonal expansion, establishing a permissive environment for leukemogenesis [29-30]. In myelodysplastic syndromes (MDS), regulatory T cells (Tregs) modify the immune landscape. Irregular toll-like receptor (TLR) signaling enhances NOD-like receptor protein 3 (NLRP3) inflammasome activity, resulting in pyroptotic cell death [18,30]. During early MDS stages, which can progress to AML, reduced CXCR4 expression in Tregs impairs their ability to control leukemic cell growth [18,20,31]. Disease progression is marked by increased Treg activity that promotes leukemic clone expansion while suppressing effective immune responses [18,30].

While T cell dysregulation and pro-inflammatory cytokines are central to RA-driven leukemogenesis, B cells also play a critical and complementary role. As shown in Table 1, B cells contribute to chronic inflammation through autoantibody production, antigen presentation, and cytokine secretion-all of which sustain the inflammatory microenvironment that fosters malignant transformation. Therefore, leukemic risk in RA arises from the interplay between dysfunctional T- and B-cell responses.

**Table 1:** Diverse roles of B cells in rheumatoid arthritis RA: rheumatoid arthritis; TET2: ten-eleven translocation 2; STAT3: signal transducer and activator of transcription 3; DNMT3A: DNA methyltransferase 3A

Function	Mechanism	Impact in RA
Autoantibody production [9]	B cells differentiate into plasma cells and produce rheumatoid factor (RF) and anti-citrullinated protein antibodies (ACPAs)	Facilitates immune complex formation, complement activation, and perpetuation of joint inflammation
Antigen presentation [10]	B cells present antigens via MHC II to CD4+ T cells	Promotes T-cell activation and polarization, contributing to chronic synovial inflammation
Cytokine secretion [7,12]	Activated B cells secrete IL-6, TNF-α, and lymphotoxin	Enhances recruitment and activation of other immune cells; promotes synovial inflammation and systemic effects
Ectopic germinal center formation [8]	B cells form aggregates in inflamed synovial tissue resembling lymphoid follicles	Sustains local autoantibody production and T-B-cell interactions
Interaction with osteoclastogenesis Pathway [2]	B cells express receptor activator of NF-κB ligand (RANKL)	Promotes osteoclast differentiation and bone erosion
Epigenetic and genetic influence [31,32]	B-cell-related mutations (e.g., in TET2, STAT3, DNMT3A) and epigenetic modifications influence B-cell behavior	May contribute to leukemogenesis and chronic RA progression

The therapeutic potential of immune checkpoint inhibitors, particularly those targeting PD-1/PD-L1, is being investigated for both leukemia and RA treatment [18,21,30]. The chronic inflammation characteristic of RA shows similarities to the inflammatory state in leukemias, indicating common disease mechanisms [18,29-30]. Current research highlights the connection between RA and increased leukemia risk, including shared epigenetic modifications such as ten-eleven translocation 2 (TET2) and DNA methyltransferase 3A (DNMT3A) mutations [17,18]. These genetic alterations influence abnormal blood cell development and could serve as potential markers for early detection and risk assessment in RA patients susceptible to leukemia development [18,30].

Distinct pathophysiological features in RA-associated leukemias 

The clinical manifestations of leukemia in RA patients vary across subtypes, with large granular lymphocyte (LGL) leukemia exhibiting a particularly distinct pathogenesis. Chronic autoantigen stimulation in RA induces sustained clonal expansion of cytotoxic T cells, particularly CD8+ T cells, which over time leads to immune dysregulation and pro-oncogenic microenvironmental changes [31]. This persistent T-cell activation promotes leukemogenesis through mechanisms such as resistance to apoptosis, increased production of pro-inflammatory cytokines (e.g., IL-15, interferon gamma (IFN-γ)), and genomic instability. Schwaneck et al. provide evidence supporting this association, although their cross-sectional study limits causal interpretation [32]. The dysregulated cytokine environment exacerbates systemic inflammation, contributing to characteristic clinical features such as polyarthritis, neutropenia, and splenomegaly, hallmarks of Felty's syndrome, which frequently coexists with LGL leukemia [33-34]. Importantly, chronic antigenic stimulation in RA may serve as a driving factor for the malignant transformation of these expanded T-cell clones, raising the possibility of T-cell leukemia or lymphoma arising as a rare but serious complication of long-standing RA.

In patients with chronic myelomonocytic leukemia (CMML) and RA, the most frequent manifestations include polyserositis and positive rheumatoid factor (autoantibody), both driven by systemic inflammation [35]. Kunnumpurath et al. rely on case reports, making the study informational but limiting generalizability. Conversely, subtypes like AML in RA patients present highly heterogeneous immune profiles where it is difficult to find shared pathways [35]. For instance, LGL leukemia patients frequently present a mutation of the signal transducer and activator of transcription 3 (STAT3) gene [36]. As these studies are developed in in vitro settings, the clinical applicability is yet to be established. Overactivation of this gene results in excessive inflammatory responses, increased cell proliferation, and apoptosis resistance [37-38].

**Figure 1:**
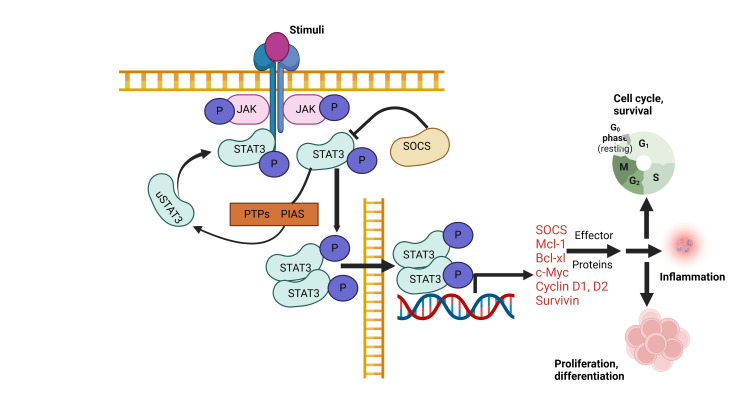
The JAK-STAT3 signaling pathway in RA-associated leukemia JAK: Janus kinase; RA: rheumatoid arthritis; STAT3: signal transducer and activator of transcription 3 Upon cytokine binding (e.g., IL-6), receptor dimerization activates JAKs, which phosphorylate STAT3. The phosphorylated STAT3 (pSTAT3) forms dimers that translocate to the nucleus, promoting transcription of genes involved in cell survival, proliferation, and inflammation, including Mcl-1, Bcl-xL, c-Myc, Cyclin D1/D2, and Survivin. While this pathway is normally regulated by SOCS proteins, PIAS, and protein tyrosine phosphatases (PTPs), chronic activation, common in RA, leads to uncontrolled immune activation, resistance to apoptosis, and malignant transformation. These molecular events underscore the role of sustained STAT3 activation in driving leukemogenesis within the pro-inflammatory milieu of RA (image credits: Khaled Aldhuaina)

In both RA and LGL leukemia, somatic mutations can occur in genes involved in hematopoiesis, including STAT3, TET2, and DNMT3A, suggesting their overlapping etiologies and highlighting the role of inflammation-driven leukemogenesis (Figure 1, Table 2) [39].

**Table 2:** Prevalent genetic mutations shared between RA and leukemia and their functional impact RA: rheumatoid arthritis; TET2: ten-eleven translocation 2; STAT3: signal transducer and activator of transcription 3; DNMT3A: DNA methyltransferase 3A; LGL: large granular lymphocyte (LGL) leukemia

Gene	Commonly implicated disease	Function of mutation	Pathophysiological effect
STAT3	RA-associated LGL leukemia	Gain-of-function mutations	Promotes T-cell clonal expansion, resistance to apoptosis, inflammation [37]
TET2	MDS, AML, RA (with CH)	Loss-of-function mutations	Epigenetic dysregulation, impaired hematopoiesis, inflammation [18]
DNMT3A	Clonal hematopoiesis, AML	Loss-of-function mutations	Disrupted DNA methylation patterns, altered lineage commitment [17,18]

Factors including disease duration, chronic exposure to antirheumatic drugs, and autoantibody profiles have been shown to influence leukemia progression. Bockorny et al. showed that autoantibodies promote clonal expansion of leukemic cells [40]. In this study, the sample size could be broader to obtain more comprehensive conclusions. Additionally, Shah et al. reported that the prevalence of T-cell clonal expansions in RA promotes the development of overt leukemia through chronic inflammation [41].

Epidemiological evidence and shared pathways linking RA and leukemias

RA demonstrates a complex relationship with hematological malignancies, driven by multifactorial causes including genetic, environmental, and autoimmune factors [42]. Luo et al. conducted a comprehensive meta-analysis of 15 studies and revealed a significantly increased leukemia risk among RA patients compared to the general population (standardized IR = 1.51; 95% CI: 1.34-1.70). Moderate statistical heterogeneity was observed (I² = 55.5%), which, based on subgroup analyses, may be attributed to variability in sample sizes across studies [42].

Epidemiological investigations have unveiled nuanced patterns of malignancy risk. Epidemiological patterns also vary regionally, with European cohorts generally showing moderate leukemia risk in RA, whereas Asian populations often exhibit higher incidence and more difficult-to-treat cases due to distinctive autoimmune and genetic predispositions [3]. Beydon et al. reported elevated hematological cancer prevalence in RA patients, documenting increased incidence of diffuse large B-cell lymphoma, follicular lymphoma, Hodgkin's lymphoma, and multiple myeloma [43]. The overall malignancy risk increases by approximately 10% compared to the general population, with heightened risks for lung cancer and lymphoma, while colorectal and breast cancer risks remain lower [44-45]. Measuring the variation in malignancy rates between RA patients treated with nonbiologic versus biologic disease-modifying antirheumatic drugs (DMARDs) could help identify which cancers are more likely linked to the treatment itself rather than the disease process [43]

Gender-specific variations add complexity to these epidemiological observations. A Chinese nationwide cohort study found that male RA patients had a significantly higher incidence of both lymphoid and myeloid leukemias, with peak risk observed between ages 61-70 for lymphoid malignancies and 51-60 for myeloid malignancies. In contrast, female RA patients demonstrated an elevated risk primarily for lymphoid malignancies [46]. The study also reported an IR of leukemia in RA patients at 0.13 per 1,000 person-years, compared to 0.08 per 1,000 person-years in matched controls, corresponding to an SIR of 1.6. These findings underscore the influence of age- and gender-related immune and biological factors in modulating leukemia risk. Hormonal differences, such as the immunomodulatory effects of estrogen, may influence B-cell activity and apoptosis pathways, potentially altering susceptibility to lymphoid malignancies in females. Conversely, male patients may experience higher rates of myeloid leukemias due to differences in immune response patterns, delayed diagnosis, or more pronounced clonal hematopoiesis. Age-related immunosenescence, cumulative inflammatory burden, and impaired DNA repair mechanisms may further elevate the risk of both lymphoid and myeloid malignancies in older adults with RA. Additionally, several mechanisms have been proposed to explain the link between autoimmune diseases and myeloid cancers, including the use of immunosuppressive therapies such as azathioprine or methotrexate (MTX), underlying genetic predispositions like IL-1 and its receptor antagonist polymorphisms, and direct bone marrow involvement or damage to myeloid precursor cells resulting from autoimmune activity.

The pathogenesis of this increased malignancy risk is multifaceted. Risk factors include MTX, antitumor necrosis factor α treatment, disease activity, and Epstein-Barr virus infection [47]. A Spanish cohort study confirmed increased frequency of developing leukemia in RA patients, associating it with disease activity, longstanding extra-articular manifestations, and cytotoxic drug use [48]. Notably, experts like John J. Cush suggest that cancer risk is more likely influenced by uncontrolled inflammation and disease activity rather than specific treatments. Patients on biologic agents appear to have similar risks to those receiving traditional DMARDs [49]. The mean duration between RA diagnosis and hematologic malignancy development ranges from 61.54 to 71.98 months [49].

The intricate relationship between RA and leukemias reveals complex immunological intersections characterized by chronic inflammatory processes, genetic predispositions, and immune system dysregulation. At the molecular level, persistent inflammatory environments play a crucial role in mediating pathogenic transitions. Pro-inflammatory cytokines, particularly IL-6 and TNF-α, demonstrate remarkable pleiotropic effects that extend beyond traditional inflammatory responses [50]. These cytokines not only perpetuate chronic immune activation but also significantly influence hematopoietic microenvironments, potentially facilitating leukemogenesis through multiple molecular mechanisms.

The bone marrow microenvironment emerges as a critical nexus where inflammatory signaling can induce profound alterations in hematopoietic stem and progenitor cell dynamics [51]. Sustained inflammatory states can promote genetic instability, epigenetic reprogramming, and cellular transformation, thereby creating permissive conditions for malignant evolution [52]. This process is further complicated by genetic and epigenetic convergence, with human leukocyte antigen (HLA) alleles representing a significant shared genetic predisposition. Specific allelic variations potentially contribute to both autoimmune susceptibility and leukemic transformation [53-54].

Lymphocyte dysfunction provides a unifying mechanism underlying these pathological processes. B-cell activation demonstrates remarkable complexity, with chronic stimulation potentially driving both autoimmune inflammation and malignant proliferation [55]. Dysregulated B-cell signaling pathways can lead to sustained immune activation, increased genetic instability, and ultimately, neoplastic transformation [56]. Similarly, T-cell pathway disruptions reveal intricate mechanisms of immune system subversion. Impaired regulatory T-cell function, altered cytokine production, and compromised immune surveillance collectively contribute to pathological conditions that blur the boundaries between chronic inflammation and malignant progression [57].

Impact of RA treatments on leukemia risk 

The relationship between RA treatments and leukemia risk is complex, and distinguishing between association and causation is critical when interpreting available evidence. While certain therapies have been linked to an increased risk of hematologic malignancies, current data often reflect correlations rather than definitive causal relationships.

MTX, a foundation drug in RA management, acts as a folate antagonist that inhibits DNA synthesis in rapidly dividing cells. Although it plays a key role in controlling disease activity, prolonged use of MTX has been associated with myelosuppression and, in rare cases, leukemogenesis, particularly at higher cumulative doses [58]. However, whether MTX directly induces leukemia or contributes to its development through long-term immunosuppression and persistent inflammation remains unresolved. Concomitant folic acid supplementation is routinely recommended with MTX to mitigate hematologic and gastrointestinal toxicity; however, current evidence does not demonstrate a significant impact on modifying long-term malignancy risk [58].

The emergence of JAK inhibitors, which disrupt key intracellular signaling pathways, has prompted renewed concern regarding hematologic malignancies. These agents may impair immune surveillance, potentially allowing premalignant clones to escape detection [59-61]. Nonetheless, current evidence is based largely on post-marketing surveillance and observational studies, and a causal link has not been definitively established.

TNF inhibitors, which target a central cytokine in RA pathogenesis, have generated conflicting findings. Some large observational studies suggest a modestly increased risk of hematologic malignancies in users of TNF inhibitors [62], while other well-controlled analyses report no significant increase, or even a possible reduction in cancer risk, attributable to the dampening of chronic inflammation [63]. These discrepancies may stem from differences in patient populations, duration of therapy, disease severity, or concurrent medication use. Thus, the potential link between TNF inhibitors and leukemia appears influenced by patient-specific factors rather than the drug alone.

In general, long-term immunosuppression, regardless of the specific therapeutic class, may contribute to leukemogenesis in genetically predisposed individuals by enabling persistent inflammation, promoting DNA damage, and reducing immunologic tumor surveillance. While biologics can reduce inflammation-driven neoplastic potential, they may simultaneously compromise immune oversight. Conversely, conventional DMARDs may modestly elevate the risk of secondary malignancies through cumulative immunosuppressive exposure, though their inflammation-modulating effects may offer protective benefits in some contexts [64].

The complexity of these treatment interactions underscores the critical need for careful patient monitoring, individualized treatment approaches, and comprehensive screening to mitigate potential long-term risks associated with RA therapies.

Clinical implications and risk stratification

**Figure 2:**
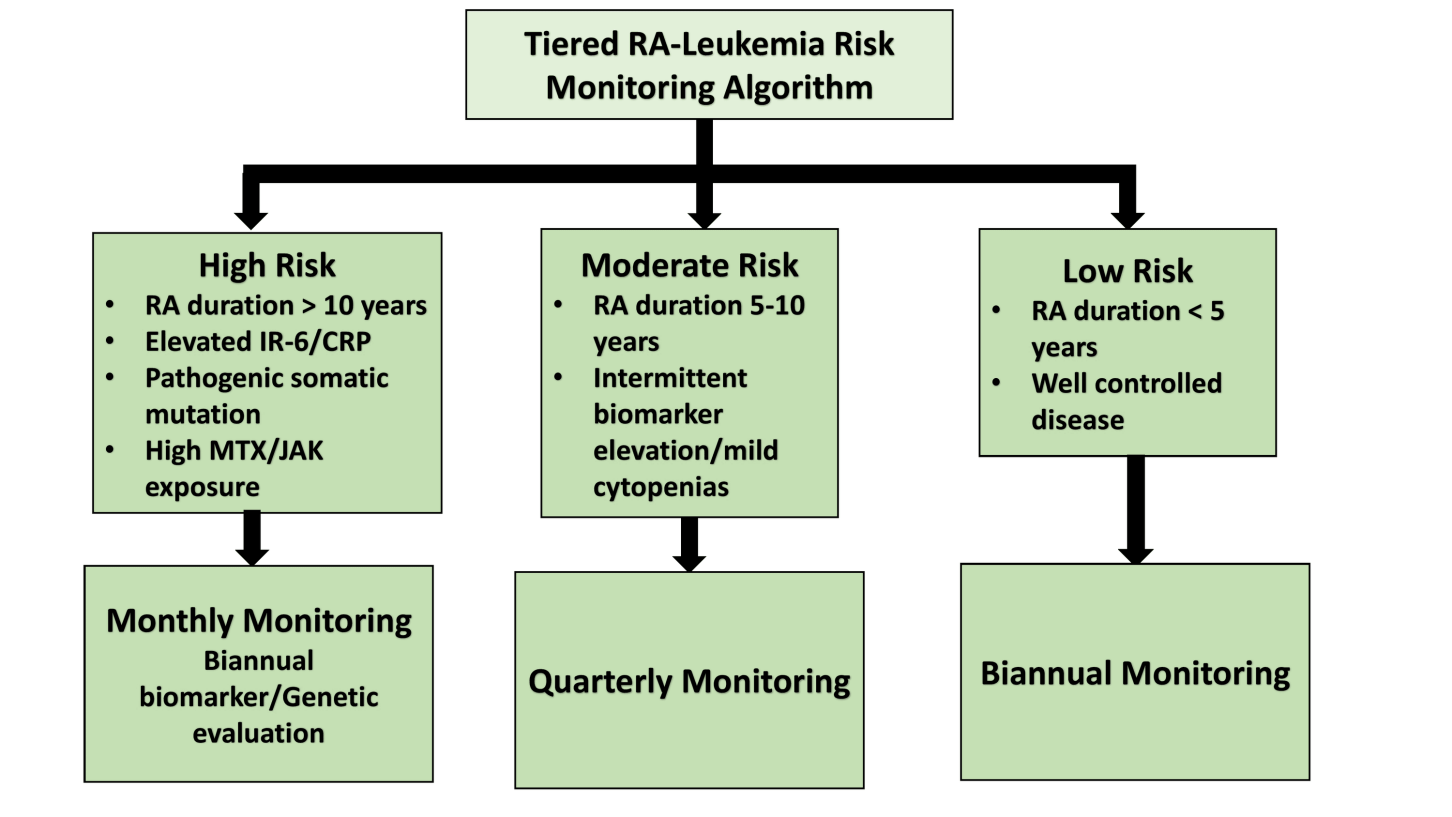
Tiered RA-leukemia risk monitoring algorithm RA: rheumatoid arthritis; MTX: methotrexate; JAK: Janus kinase; IL-6: interleukin-6; CRP: C-reactive protein; This flowchart outlines a structured risk-stratification model for monitoring leukemia risk in patients with RA. Patients are categorized into three tiers based on disease duration, biomarker levels (e.g., IL-6, CRP), genetic findings, and treatment exposures (image credits: Aakriti Datta)

The identification of high-risk individuals necessitates comprehensive biomarker evaluation (Figure 2). Elevated pro-inflammatory cytokine profiles, particularly IL-6, serve as potential predictive indicators for leukemogenesis. Early detection strategies encompass genetic screening for pathogenic mutations, monitoring for neutropenia, and surveillance of hematological parameters for cytopenias or unexplained leucocytosis [65]. Critical screening modalities include systematic hematological assessment, biomarker analysis, advanced imaging techniques (MRI and PET scans), and bone marrow evaluation. Integration of these proactive screening protocols into standard RA care pathways is essential for early detection and intervention [66].

Implementation of an algorithm-based risk stratification system can optimize resource allocation and patient monitoring. Patients can be categorized into risk tiers (high, medium, or low) based on clinical parameters and biomarker profiles [67-68]. To operationalize this, a tiered risk-stratification algorithm is proposed wherein high-risk patients include those with long-standing RA (greater than 10 years), persistently elevated inflammatory biomarkers such as IL-6 and CRP, presence of pathogenic somatic mutations, prior exposure to high cumulative doses of MTX or JAK inhibitors, or existing cytopenias. These individuals warrant monthly hematologic assessments, biannual genetic and biomarker evaluations, and close coordination with hematology. The moderate-risk group comprises patients with intermediate disease duration (5-10 years), intermittent biomarker elevations, or mild cytopenias, for whom quarterly monitoring is recommended. Patients in the low-risk tier, those with early-stage RA (less than five years), well-controlled disease, and no hematologic or genetic abnormalities, can be adequately monitored on a biannual basis [69]. Widely used composite indices such as DAS28 and CDAI may also aid in risk stratification, as higher disease activity scores have been associated with increased systemic inflammation and potentially greater susceptibility to hematologic malignancies [67].

Interdisciplinary coordination is critical but fraught with practical challenges. Treatment objectives between specialties may diverge: rheumatologists prioritize disease remission through immunosuppression, while hematologists may require immune system preservation for effective leukemic control. For instance, high-dose immunosuppressive therapies (e.g., biologics or JAK inhibitors) may conflict with leukemia treatment protocols due to increased infection risk or bone marrow suppression.

Moreover, treatment regimens often have overlapping toxicities, MTX and cytotoxic chemotherapies both carry myelosuppressive risks, necessitating dose adjustments and careful sequencing. Coordinated care is facilitated by multidisciplinary team meetings, integration of treatment plans via shared electronic health records (EHR), and real-time communication across specialties [68]. Implementation of this structured algorithm and acknowledgment of treatment incompatibilities allow for individualized patient-centered care that balances efficacy with safety in managing coexisting RA and leukemia.

Future research directions 

The complex relationship between RA and leukemias presents a compelling opportunity to advance our understanding of shared pathophysiological mechanisms through longitudinal epidemiological investigations [38]. While established evidence supports the association between chronic inflammation and immunological dysregulation in RA with elevated leukemia risk, the precise molecular mechanisms remain incompletely understood [69]. Current literature predominantly comprises retrospective and cross-sectional analyses, which, while valuable, establish correlative rather than causal relationships [70]. Disease activity assessment in RA using inflammatory markers such as C-reactive protein (CRP) and erythrocyte sedimentation rate (ESR) provides essential insights into disease severity and potential leukemia risk. Furthermore, documentation of therapeutic exposures-particularly DMARDs, TNF inhibitors, and corticosteroids-is vital for evaluating their role in leukemogenesis [5,69]. Enriching future research cohorts with patients having long-standing RA, severe clinical manifestations, or a family history of hematologic malignancies could improve study relevance and power [71].

Advances in biomarker discovery offer a particularly promising direction for research at the intersection of RA and leukemia. Contemporary molecular profiling approaches, including transcriptomics and epigenomics, have identified disease-specific markers that can aid in early detection and risk stratification. Machine learning approaches have uncovered distinct gene expression profiles that reliably separate RA patients from healthy controls. Multiomics integration has successfully validated potential biomarkers such as CRTAM, PTTG1IP, MMP13, and ITGB2. Of these, CRTAM is notably implicated in multiple malignancies, underscoring its value in RA-associated leukemogenesis. These molecular insights highlight the potential of biomarker-guided surveillance in high-risk RA patients. Moreover, AI-driven risk models incorporating cytokine profiles, somatic mutations, and epigenetic data may significantly enhance personalized disease monitoring and targeted intervention strategies [69,72-73].

Current therapeutic agents, including JAK inhibitors, CD20 monoclonal antibodies, and immune checkpoint modulators, continue to play pivotal roles in RA management and may also exert protective effects against leukemic transformation when appropriately selected. Shared molecular targets between RA and specific leukemia subtypes, such as those seen in large granular lymphocyte leukemia, present novel opportunities for dual-purpose therapeutic development [69,74-75]. Future studies should also explore establishing evidence-based recommendations on limiting the cumulative dose or treatment duration of RA therapies with known leukemogenic potential, particularly in high-risk patients, to balance disease control with long-term safety. Advancing these translational goals will require robust interdisciplinary collaboration among rheumatologists, hematologists, immunologists, and molecular researchers. Coordinated, multi-specialty research is essential to close current knowledge gaps and develop integrated preventive, diagnostic, and therapeutic frameworks for patients affected by both RA and hematologic malignancies.

## Conclusions

The intricate relationship between RA and leukemias underscores the significant overlap in inflammatory pathways, genetic predispositions, and immune dysregulation that contribute to disease progression. Chronic inflammation, driven by pro-inflammatory cytokines such as TNF-α and IL-6, not only fuels joint destruction in RA but also fosters a permissive environment for leukemogenesis. Epidemiological data highlight an increased risk of hematological malignancies in RA patients, necessitating vigilant monitoring and risk stratification. Furthermore, the impact of RA treatments, particularly immunosuppressive agents, presents a dual challenge in balancing disease control with malignancy risk. Advancing research through multiomics approaches, biomarker discovery, and interdisciplinary collaboration is crucial for developing precision medicine strategies that mitigate leukemia risk while optimizing RA management. Enhanced surveillance, early detection protocols, and targeted therapeutic interventions hold promise for improving outcomes in this high-risk patient population.
